# Systematic analysis of the thioredoxin gene family in *Citrus sinensis*: identification, phylogenetic analysis, and gene expression patterns

**DOI:** 10.1080/15592324.2023.2294426

**Published:** 2023-12-17

**Authors:** Xiru Zuo, Cheng Yang, Yana Yan, Guiyan Huang, Ruimin Li

**Affiliations:** College of Life Sciences, Gannan Normal University, Ganzhou, China

**Keywords:** *Citrus sinensis*, thioredoxin, expression, subcellular localization, redox

## Abstract

Thioredoxin (TRX) proteins play essential roles in reactive oxygen species scavenging in plants. We executed an exhaustive analysis of the TRX gene family in *Citrus sinensis* (*CsTRXs*), encompassing identification, phylogenetic analysis, detection of conserved motifs and domains, gene structure, cis-acting elements, gene expression trends, and subcellular localization analysis. Our findings established that a total of 22 *CsTRXs* with thioredoxin domains were identified in the genome of *C. sinensis*. Phylogenetic analysis indicated that CsTRXs were divided into six subclusters. Conserved motifs analysis of CsTRXs indicated a wide range of conserved motifs. A significant number of cis-acting elements associated with both abiotic and biotic stress responses, inclusive of numerous phytohormone-related elements, were detected in the promoter regions of *CsTRXs*. The expression levels of *CsTRXs* including *CsTRXf1*, *CsTRXh1*, *CsTRXm1*, *CsTRXo3*, *CsTRXx2* and *CsTRXy1* were observed to be reduced upon pathogen infection. Subcellular localization analysis found that CsTRXf1, CsTRXm1, CsTRXo3, CsTRXx2 and CsTRXy1 were predominantly localized in chloroplasts, whereas CsTRXh1 was distributed indiscriminately. This research yields integral data on *CsTRXs*, facilitating future efforts to decipher the gene functions of *CsTRXs*.

## 1. Introduction

Citrus fruits, grown in over 114 countries, are highly prized for their rich vitamin C content, a potent antioxidant that bolsters the human immune system.^1,2^ Regrettably, Huanglongbing (HLB) has wrought devastating damage on citrus production, precipitating a downfall in crop yield, nutritional merit, and market quality.^3^ HLB is incited by the phloem-dwelling bacterium *Candidatus* Liberibacter asiaticus (CLas). While CLas is not directly responsible for HLB symptoms due to its deficiency of pathogenicity factors, it can nevertheless trigger immune responses and cellular apoptosis in phloem tissue.^4,5^ CLas infection instigates symptoms such as leaf mottling, twig dieback, undersized and asymmetrical fruits, and root decay.^5,6^ Regrettably, the challenge of *in vitro* cultivation of CLas has impeded research into its pathogenicity. It has been evidenced that CLas infection elevates the expression of NADPH oxidases genes, thus escalating the generation of reactive oxygen species (ROS) and diminishing the expression of antioxidant enzyme-coding genes.^5,7^ These findings imply that CLas incites oxidative stress, and the ROS levels prompted by CLas can achieve a magnitude capable of inducing cell death, particular to companion and sieve element cells of mature leaves. Remarkably, administering antioxidants to CLas-infected citrus leaves has shown to decrease hydrogen peroxide (H_2_O_2_) content and cell death in phloem tissues, consequently mitigating HLB symptoms.^5^ Furthermore, it is established that plant growth is inhibited when exposed to 1 mM H_2_O_2_, which could elucidate the growth retardation phenotype observed in young citrus trees afflicted by CLas.^8^

TRXs are universally conserved proteins, observable across a diverse range of organisms.^9^ Endowed with two cysteine residues within its catalytic site, TRX forms a disulfide bond targeting and reducing substrate proteins, thereby enabling its participation in cellular redox reactions.^10,11^ TRX is implicated in numerous physiological and biochemical phenomena, from gene expression, redox homeostasis, and apoptosis to hormone transport and environmental responses.^12^ By modulating the redox equilibrium of their active site’s sulfhydryl group, TRX proteins can transmit hydrogen and electrons. This makes them instrumental in managing photosynthesis, substrate metabolism in fluctuating light conditions, and metabolic flux through the tricarboxylic acid cycle.^13^ Moreover, TRX systems are key to maintaining cellular redox balance and managing stress responses within redox biochemical pathways. Higher plants feature an extensive gene family encoding for TRX, contrasting the two isoforms found in other organisms like mammalian cells.^14^ Based on sequence similarity and subcellular localization, plant TRX proteins are divided into six categories. These categories include TRXf, TRXm, TRXx, and TRXy found in the chloroplast, and TRXh, typically discovered in the cytoplasm and reduced by NADPH-dependent TRX reductases in mitochondria or cytoplasm.^10,15^ Plants exhibit a highly evolved TRX system, as evidenced by the 61 TRXs in rice, 86 in cotton, and 48 in grape.^15–17^ These proteins are categorized as typical or atypical based on their active site variations. Typical TRXs usually embody a conserved Cys-Gly-Pro-Cys (CGPC) motif at the N-terminus, while atypical ones include other conserved motifs such as CPHC in protein disulfide isomerase (PDI) and CGHC in disulfide oxidoreductase.^10,18^ For instance, the grape genome comprises 48 TRX members, with 18 exhibiting the typical “CXXC” TRX motif, 18 depicting an atypical variant of this motif, and the rest lacking the conserved motif.^15^ Similarly, of the 86 TRXs present in the cotton genome, 40 are typical, and the remaining 46 are atypical.^17^

The TRX family has been found to be integral to plant development and adaptation to environmental stressors. For instance, *AtTRXf1*, upon analysis, was found to be involved in the allocation of carbon for photosynthesis.^19^ A significant reduction in plant growth, alterations in light acclimation, and a substantial decrease in Calvin-Benson-cycle activity and starch accumulation were observed in a double-deficient mutant of *TRXf1* and *NTRC*, a phenomenon not seen in single deficient mutants in *Arabidopsis*.^20^ TRXh9, a plasma membrane protein in Arabidopsis, has been implicated in intercellular communication, with aberrant gene expression leading to abnormal plant development.^21^ Multifunctional TRXm4 has been linked to the inhibition of cyclic electron transport in chloroplast photosystem I.^22^ Notably, a member of the TRX family in *Vitis vinifera*, categorized under subgroup IV in *TRXh*, is associated with ovule abortion in seedless grapes.^23^ Mitochondrial thioredoxins are believed to modulate apoptosis by modifying the conformation of porin.^24^ Plants have developed sophisticated defense mechanisms in response to pathogenic and herbivorous attacks, with TRX showing reactivity to plant immune responses. For instance, *ZmTRXh* demonstrates a unique defense response during the early stages of maize resistance to the Sugarcane mosaic virus.^25^ *MaTRX12* is reported to enhance the low-temperature tolerance of banana fruit by managing redox homeostasis.^26^ In rice, salt stress triggers the secretion of OsTRXh1 in the apoplast, and the absence of OsTRXh1 protein results in increased H_2_O_2_ levels, leading to stunting and low tillering during the later stages of plant development.^27^

To gain insight into the roles of *TRX* genes in CLas infection, a comprehensive examination of the *CsTRXs* was performed. The *CsTRXs* were identified and annotated in the genome of *C. sinensis*. Subsequent analysis of this gene family included assessing phylogenetic relationships, chromosome distribution, conserved motif analysis, gene structure analysis, and types of cis-acting elements. Further, the expression patterns of candidate *CsTRXs* and subcellular localization were explored. This research provided vital information about the expression of various *CsTRXs* under CLas infection and set a foundation for future research into the biological function and regulatory mechanisms of *CsTRXs*.

## 2. Materials and Methods

### 2.1. *Identification and sequence analysis of* CsTrxs

The complete genome sequence of *C. sinensis* was retrieved from CPBD (Citrus Pan-genome to Breeding Database),^28^ and the Hidden Markov Model (HMM) file of TRX conserved domain (Thioredoxin.hmm [PF00085]) was downloaded from the Pfam database.^29^ Using ‘Thioredoxin.hmm’ as the index file, HMMER 3.0 software was used to identify potential *CsTRXs*.^30^ The identified *CsTRXs* were further verified by uploading the sequences to NCBI CDD database and SMART online tools.^31,32^ The molecular weight (MW) and isoelectric point (PI) of the CsTRXs were predicted using “Compute pI/Mw tool”.^33^ The grand average of hydropathicity (GRAVY) of the CsTRXs were predicted using “ProtParam tool”.^33^ Moreover, the subcellular localization of the CsTRXs were predicted using Plant-mSubP.^34^

### 2.2. Phylogenetic analysis and multiple sequence alignment

The protein sequences of identified TRXs in *A. thaliana* were obtained from The *Arabidopsis* Information Resource database (TAIR).^35^ Phylogenetic analysis of CsTRXs and AtTRXs was conducted using the analysis process provided by PhyloSuite software with alignment using MAFFT, model selection using ModelFinder and phylogenetic tree construction using IQ-tree.^36–39^ The conserved domain sequence was extracted, and the protein sequences were aligned through CLC sequence viewer 8.0 software with default parameters. Additionally, conserved sequence logo analysis was fulfilled using the WebLogo online tools.^40^

### 2.3. Chromosomal location and gene structure analysis

Utilizing the gene structure annotation information of *CsTRXs* obtained from CPBD, TBtools “Gene Location Visualize from GTF/GFF” module was employed to visualize the chromosomal location of *CsTRXs*.^41^ Additionally, the intron – exon structures of *CsTRXs* were determined using TBtools “Visualize Gene Structure” module.^41^

### 2.4. Conserved domain and conserved motif analysis

The identification of conserved domains among CsTRXs were conducted using the NCBI CDD database,^31^ and the MEME online tool^42^ was utilized to analyze the conserved motifs. The parameters for the tool were set to 10 modules, with a minimum module length of 6 and a maximum module length of 50, while all other parameters kept as default.

### 2.5. Cis*-acting elements analysis*

To generate the *cis*-acting elements in the upstream regions of *CsTRXs*, the 2000 bp upstream sequences of the *CsTRXs* initiation codon were isolated from the reference genome of *C. sinensis*.^28^ the PlantCARE database was consulted for any associated cis-acting elements.^43^ The TBtools software was then utilized to display the distribution of biotic and abiotic response elements.^41^

### 2.6. Expression analysis by qRT-PCR

The two years old CLas-free and -infected *C. sinensis* Osbeck cv. ‘Newhall’ plants cultivated in artificial climate room were used as experimental materials, and the total RNA was extracted using the EasyPure® Plant RNA Kit (Transgen, China) and reverse transcribed with the TransScript® One-Step gDNA Removal and cDNA Synthesis SuperMix (Transgen, China). Then the real-time quantitative PCR procedure was conducted on an ABI step one PCR instrument. Six candidates *CsTRXs*, namely *CsTRXh1*, *CsTRXf1*, *CsTRXm1*, *CsTRXy1*, *CsTRXx2* and *CsTRXo3*, were selected and qRT-PCR was applied to detect their expression levels with the designed primers (Table S1). GAPDH in *C. sinensis* was used as internal reference gene in this study and 2^−ΔΔCT^ method was used to calculate the expression of candidate genes.^44^

### 2.7. Subcellular localization analysis

Six candidates *CsTRXs*, namely *CsTRXh1*, *CsTRXf1*, *CsTRXm1*, *CsTRXy1*, *CsTRXx2* and *CsTRXo3*, were selected for subcellular localization analysis. The coding sequences of the six candidate *CsTRX* genes with the stop codons removed were inserted into the plant binary vector pCAMBIA2300-GFP and fused with GFP using restriction enzymes of *Sac* I and *Xba* I. Then the recombinant vectors were transformed into the *Agrobacterium tumefaciens* GV3101. Subsequently, the *A. tumefaciens* strains carrying recombinant vectors were cultivated and suspended with MES buffer as described in previous study,^45^ respectively. The suspended bacteria solutions were injected into the leaf epidermal cells of *Nicotiana benthamiana* and cultivated for 2 days. Then the subcellular localization was observed using a Leica TCS-SP8 confocal microscopy.

### 2.8 Statistics analysis

The experimental data’s statistical significance was assessed using SPSS 25.0 and analyzed through Student’s unpaired two-sided *t*-test.

## 3. Results

### 3.1. *Identification and sequence analysis of the* CsTrxs

HMMER software was employed to identify proteins containing the thioredoxin domain in *C. sinensis* genome. Combining the NCBI CDD database and SMART online prediction tools, a total of 22 members of *CsTRXs* in *C. sinensis* were identified. Analysis of the physical and chemical properties of *CsTRXs* revealed that the coding region of *CsTRXs* varied in length, with *CsTRXh5* being the longest of 1,047 bp and *CsTRXh1* being the shortest of 363 bp (Table S2). The protein length of CsTRXs ranged from 120 to 348 amino acids, with the corresponding molecular weight ranging from 13.21 to 39.02 KDa and the isoelectric point ranging from 4.45 to 10.29 (Table S2). Additionally, the hydrophilicity of CsTRXs ranged from −0.642 to 0.091. Predicted subcellular location analysis showed that the majority of CsTRXs were located in mitochondria and chloroplasts, with a minority in the nucleus (Table S2).

### 3.2. Phylogenetic and conserved domain analysis of the CsTrxs

**Figure 1.**
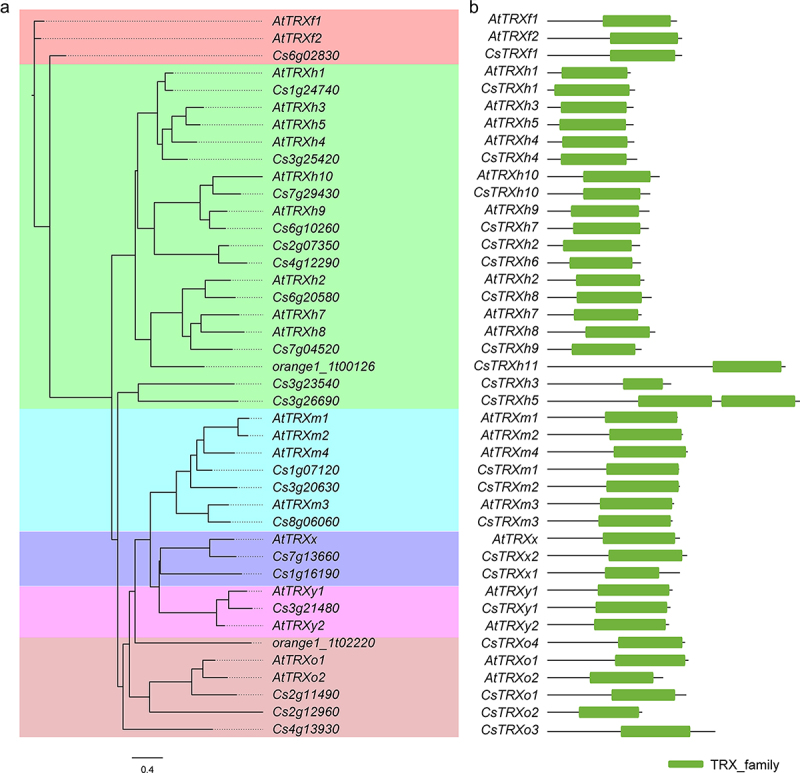
Phylogenetic and conserved domain analysis of thioredoxin (TRX) members identified in *Arabidopsis thaliana*, *Citrus sinensis* genomes. (a) phylogenetic analysis; (b) conserved domain exhibition. Sequence alignment was conducted using MAFFT. The phylogenetic tree was constructed using the maximum likelihood method by IQ-tree.

A phylogenetic tree was constructed using the maximum likelihood method by IQ-tree to elucidate the classification and evolutionary relationships among CsTRXs and AtTRXs using protein sequences. Phylogenetic results indicated the CsTRXs and AtTRXs could be divided into six distinct clades, which is in accordance with previous studies (Figure 1a). Specially, 1 TRXf, 11 TRXhs, 3 TRXms, 4 TRXos, 2 TRXxs and 1 TRXy were identified in *C. sinensis* and the genes were named base on their location information on the chromosomes (Figure 1b). all the identified CsTRXs contained one or two conserved thioredoxin domain at the C terminal (Figure 1b).

### 3.3. *Chromosomal location of the* CsTrxs

**Figure 2.**
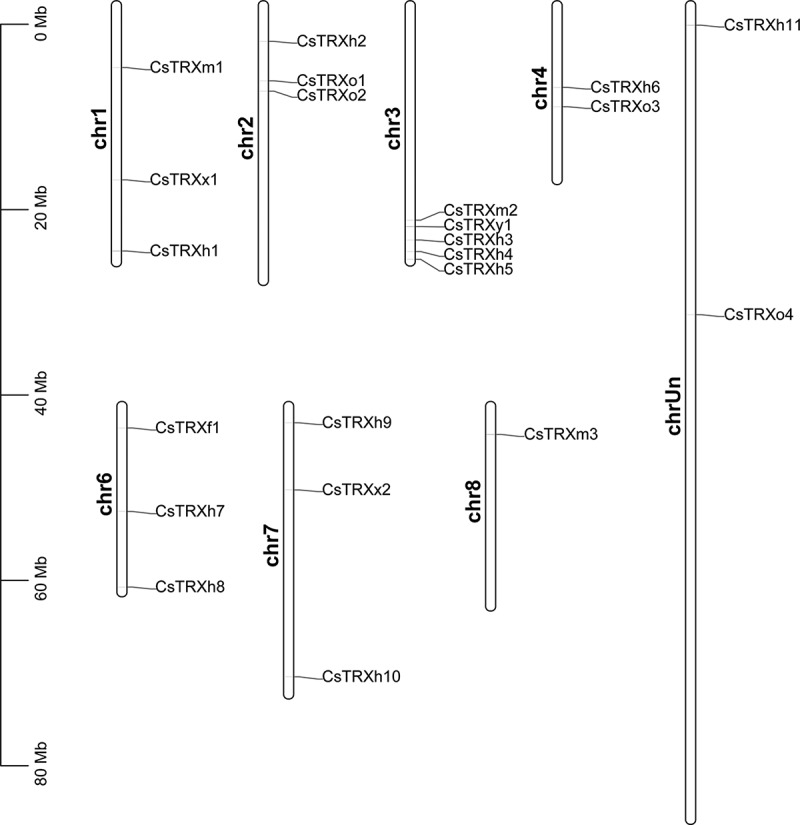
Chromosomal location analysis of *CsTrxs* in *Citrus sinensis*.

To understand the chromosomal location, TBtools were utilized to display the distribution of *CsTRXs* on the chromosomes.^41^ Results indicated that the distribution of the 22 *TRXs* in *C. sinensis* was not uniform, as evidenced by their presence across 7 different chromosomes (Figure 2). Notably, chromosome 3 exhibited the highest number of *TRX* genes, with a total of 5. Chromosomes 1, 2, 6, and 7 each harbored 3 *TRX* genes, while chromosomes 4 and 8 contained 2 and 1 *TRX* gene, respectively (Figure 2). Additionally, *CsTRXh11* and *CsTRXo4* were not found on the known citrus chromosome due to the imperfection of the assembled reference genome, thus they have been assigned to the chrUn chromosome (Figure 2).

### 3.4. *Gene structure analysis of* CsTrxs

**Figure 3.**
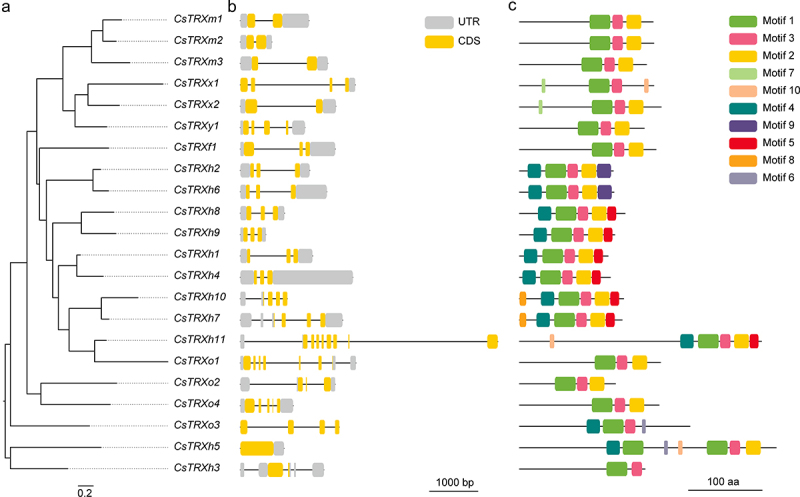
Gene structure and conserved motif analysis of *CsTRXs*. (a) phylogenetic analysis; (b) gene structure analysis; (c) conserved motif analysis. The phylogenetic tree was constructed using the maximum likelihood method by IQ-tree.

Furthermore, the gene structures were studied to uncover the exon-intron structure of *CsTRXs* (Figure 3). The exon-intron structure of the 22 *CsTRXs* showed a structural diversity, with the number of exons ranging from 1 to 8 (Figure 3b). Thereinto, *CsTRXh11* had the highest number of exons (8), followed by *CsTRXo1* (6), and *CsTRXh5* having the least (1). Interestingly, the number of exons among genes with greater sequence similarity remained consistent (Figure 3b).

### 3.5. Conserved motifs and conserved sites in the CsTrxs

**Figure 4.**
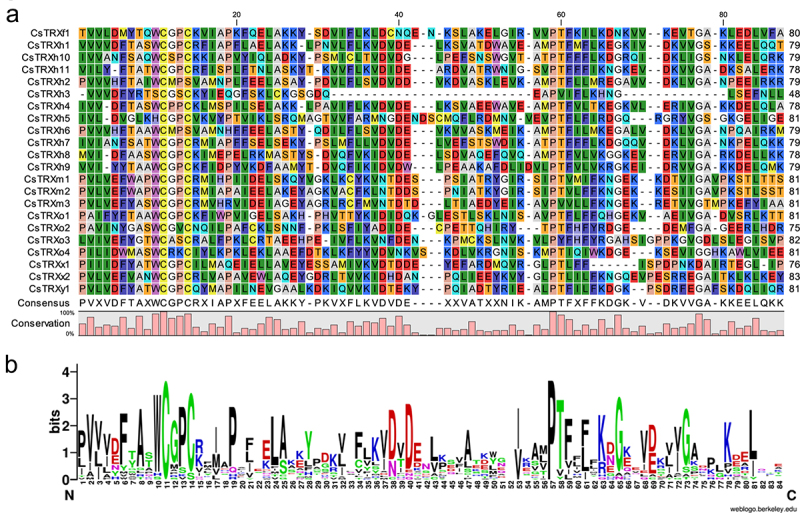
Multiple sequences alignment and conserved amino acids logo analysis of CsTRXs. (a) multiple sequences alignment; (b) conserved amino acids logo analysis.

Analysis of the conserved motifs of CsTRXs using the online software MEME revealed that there were 10 conserved motifs among the various family members, with some motifs being shared by all members and others being exclusive to certain members (Figure 3c). For instance, Motif 1 and Motif 3 were found in all CsTRXs, while Motif 7 and Motif 8 were only present in CsTRXx1 and CsTRXx2, and CsTRXh10 and CsTRXh7, respectively. Additionally, CsTRXh5 contained the highest number of conserved motifs (7). Subsequently, a multiple sequences alignment of the conserved TRX domains were conducted using CLC sequence viewer (Figure 4), resulting in the identification of 11 conserved amino acid sites.

### 3.6. *Analysis of cis-acting elements of the* CsTrxs

This study utilized the PlantCare database to analyze the promoter sequences (2,000 bp) upstream of the coding sequences to identify 13 categories of cis-acting elements related to stress and phytohormone response. Of the 577 cis-acting elements identified in 22 *CsTRXs*, 236 were classified as MYB binding sites, the most abundant type. The other categories included Auxin-responsiveness, Ethylene-responsiveness, Gibberellin-responsiveness, MeJA-responsiveness, Salicylic acid-responsiveness, WRKY binding site, Defense and stress-responsiveness, Drought and high-salinity stress responsiveness, Low-temperature-responsiveness, and Wound-responsiveness (Figure S1). *CsTRXh4* and *CsTRXm2* had the highest and lowest number of cis-acting elements, respectively, with 52 and 12 (Figure S1).

### 3.7. *Expression profiles analysis of* CsTrxs

**Figure 5.**
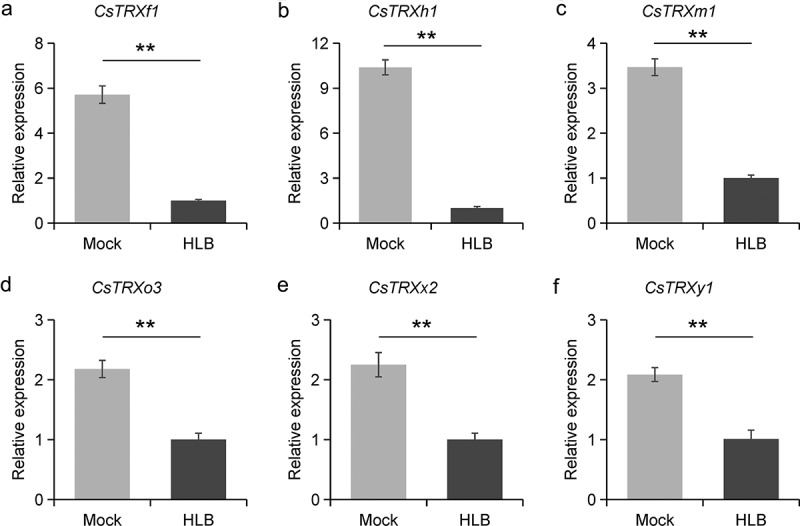
Expression profiles analysis of *CsTrxs* during CLas infection. (a) *CsTRXf1*; (b). *CsTRXh1*; (c) *CsTRXm1*; (d). *CsTRXo3*; (e) *CsTRXx2*; (f). *CsTRXy1*. Mock indicated CLas free citrus plants and HLB indicated CLas infected citrus plants. Error bars indicate the standard deviation of three biological replicates, with a significance of p-value < .01 indicated by ”**”.

The expression profiles of *CsTRXs* in CLas-free and -infected *C. sinensis* were determined by qRT-PCR. In response to CLas infection, the expression levels of all six candidates *CsTRXs*, namely *CsTRXh1*, *CsTRXf1*, *CsTRXm1*, *CsTRXy1*, *CsTRXx2* and *CsTRXo3*, were significantly decreased (Figure 5). It is noteworthy that the expression level of *CsTRXh1* in samples CLas-infected was 10.35-fold lower than in CLas-free samples of *C. sinensis* (Figure 5b).

### 3.8. Subcellular localization analysis of CsTrxs

**Figure 6.**
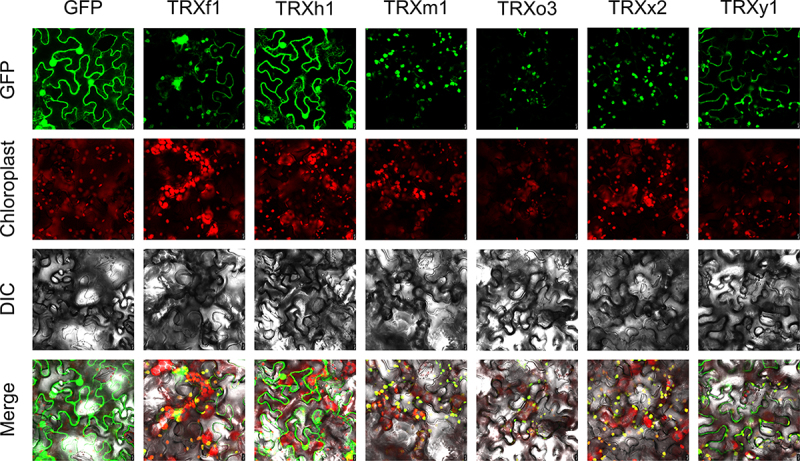
Subcellular localization analysis of CsTRXs. Free GFP was used as a control. Scale bar, 10 μm.Frefe.

To determine the subcellular localization of CsTRXs, six candidates *CsTRXs* were transient expression in the leaves of *N. benthamiana*. Results indicated that CsTRXf1 and CsTRXy1 were predominantly located in chloroplasts, though they were also present in the cytoplasm and cytomembrane. However, CsTRXh1 was distributed in all organelles, while CsTRXm1, CsTRXo3 and CsTRXx2 were mainly found in chloroplasts (Figure 6).

## 4. Discussion

Despite their significance, there is a paucity of reports on the sequence data and biological roles of TRX in *C. sinensis*. In the current study, we performed a thorough examination of the *CsTRXs* in *C. sinensis*, encompassing phylogenetic analysis, investigation of conserved motifs and domains, gene structure, cis-acting elements, gene expression patterns, and subcellular localization. Importantly, we have compared gene expression patterns in CLas-free and -infected samples.

Current studies reveal a substantial disparity in the quantity of *TRX* genes amongst diverse plant species.^15,17^ In the genome of *C. sinensis*, 22 *CsTRXs* were identified. Typical TRXs contain a conserved “CGPC” motif at the N-terminus, whereas atypical TRXs display other conserved motifs such as “CXXC”. Out of the 48 TRX genes in the grape genome, 18 are typical TRXs, 18 are atypical TRXs, and the remaining 12 do not contain the “CXXC” motif.^15^ Upland cotton comprises 40 typical TRXs and 46 atypical TRXs.^17^ In this study, 15 of the identified 22 *CsTRXs* were typical, and 7 were atypical (Table S2). The differences between typical and atypical TRXs, particularly at the key amino acid positions of their active centers, could influence their functions given the broad involvement of TRXs in various biological processes.

The expression levels of *CsTRXs* are regulated by *cis*-acting elements in the promoter regions. The distribution of abiotic and biotic stresses responsive elements is not uniform across different promoter regions; for instance, *CsTRXh4* contains 52 *cis*-acting elements, whereas *CsTRXm2* has only 12 (Figure S1). It is believed that MYB transcript factors have a major influence on the regulation of *CsTRXs*, and this is largely due to the abundance of MYB binding sites, the most common type of *cis*-acting elements.^46^ In addition, Salicylic acid-responsiveness, WRKY binding site, and Defense and stress-responsiveness elements may also be involved in the process of citrus-pathogen interactions as TRXs respond to plant immune reactions.^47^ For instance, GbNRX1 in island cotton maintains intracellular stability during a cotton wilt disease outbreak by quickly balancing the redox level.^48^ NtTRXh3 in tobacco plants enhances resistance to tobacco mosaic virus and cucumber mosaic virus.^49^ The present study investigated the response of *CsTRXs* to CLas infection. Interestingly, all selected candidates showed down-regulated during CLas infection by qRT-PCR. However, previous studies demonstrated that some *CsTRXs* were up-regulated during CLas infection.^50,51^ One possible reason is due to the use of different citrus varieties or variations in the timing of CLas infection.

The localization of proteins within the cell is indispensable for performing their functions. Our study initially predicted the subcellular localization of CsTRXs using Plant-mPLoc (Table S2), and then experimentally verified six selected candidates (Figure 6). The CsTRXh1 were predicted to be localized in cytoplasm while the CsTRXh1 was found to be distributed in all organelles including nucleus, cytoplasm (Figure 6). Research has demonstrated that f, m and x type TRXs are located in chloroplasts^52^ and our findings are in agreement with the results. Previous studies indicated that NbTRXh2, a h type thioredoxin, localized at the plasma membrane. However, in our study, the h type thioredoxin, CsTRXh1, localized not specificity. The subcellular localization results indicated that f, m, o, x and y type thioredoxins mainly participant in the chloroplast system while the h type thioredoxin may functional diversity.

## 5. Conclusions

In the present study, we conducted an extensive examination of the *CsTRXs* in *C. sinensis*, including phylogenetic analysis, identification of conserved motifs and domains, gene structure assessment, cis-acting elements identification, gene expression pattern, and subcellular localization analysis. We identified 22 *CsTRXs* containing thioredoxin domains within the *C. sinensis* genome, which were further categorized into six subclusters. The promoter regions of *CsTRXs* contained numerous response elements for both abiotic and biotic stress, inclusive of several phytohormone-related cis-acting elements. The CLas infection was found to mitigate the expression levels of *CsTRXs*. Notably, CsTRXf1, CsTRXm1, CsTRXo3, CsTRXx2, and CsTRXy1 were predominantly localized in chloroplasts, while CsTRXh1 displayed widespread distribution. The detailed information on *CsTRXs* in *C. sinensis*, obtained from our study, might prove beneficial for further functional analysis of these genes.

## Supplementary Material

Figure S1.tif

Table S1.docx

Table S2.docx

## Data Availability

All the data relevant to this study are included in the article or uploaded as Supplementary Materials.
