# Supplementary figures and images for: Systematic analysis of the thioredoxin gene family in Citrus sinensis: identification, phylogenetic analysis, and gene expression patterns
Source: Plant Signal Behav. 2023 Dec 17;18(1):2294426. doi: 10.1080/15592324.2023.2294426 (PMC10730155; doi:10.1080/15592324.2023.2294426)

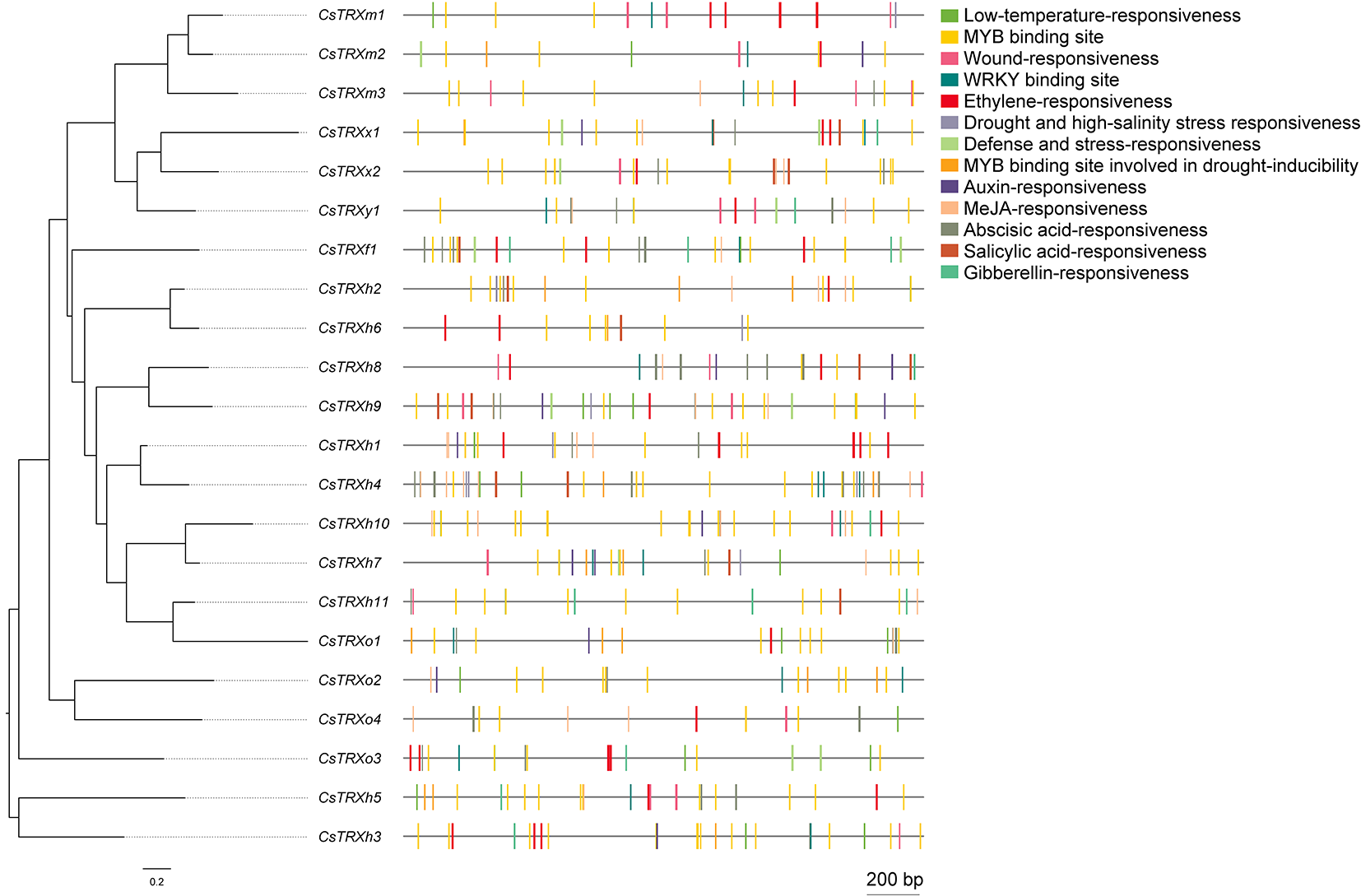

Supplement: Figure S1.tif [file KPSB_A_2294426_SM1107.tif]
