# Supplementary material for: Systematic analysis of the thioredoxin gene family in Citrus sinensis: identification, phylogenetic analysis, and gene expression patterns
Source: Plant Signal Behav. 2023 Dec 17;18(1):2294426. doi: 10.1080/15592324.2023.2294426 (PMC10730155; doi:10.1080/15592324.2023.2294426)
Supplement: Table S1.docx [file KPSB_A_2294426_SM1106.docx]

Table S1. Primers used in this study.

| Prime name | Prime sequences | Usage | Locus ID |
| --- | --- | --- | --- |
| CsTRXh1-2300-F | AGAACACGGGGGACGAGCTCATGGCAGCAGCAGAAGAGGG | Vector construction | Cs1g24740 |
| CsTRXh1-2300-R | ACCATGGTGTCGACTCTAGAGGCAGAGGCAGTTGCCAG |  |  |
| CsTRXf1-2300-F | AGAACACGGGGGACGAGCTCATGTCTTTACTCCAATTCTCCTCCACC |  | Cs6g02830 |
| CsTRXf1-2300-R | ACCATGGTGTCGACTCTAGAACCGGATCTAACAGCATCAATGG |  |  |
| CsTRXm1-2300-F | AGAACACGGGGGACGAGCTCATGGCCACCGTGCTCGAGTG |  | Cs1g07120 |
| CsTRXm1-2300-R | ACCATGGTGTCGACTCTAGACAAGAATTTCTCTATGCTGGTGGTC |  |  |
| CsTRXo3-2300-F | AGAACACGGGGGACGAGCTCATGGCAGATGTTATAAGGGCATG |  | Cs4g13930 |
| CsTRXo3-2300-R | ACCATGGTGTCGACTCTAGAACTTGATCCTGCCGGCTTCTCC |  |  |
| CsTRXx2-2300-F | AGAACACGGGGGACGAGCTCATGGACATCGTCTTCTCCAACTCC |  | Cs7g13660 |
| CsTRXx2-2300-R | ACCATGGTGTCGACTCTAGAAGCCACTGAGATTGAATCTAAAAGAG |  |  |
| CsTRXy1-2300-F | AGAACACGGGGGACGAGCTCATGGCGATTTCTTTGACGGCG |  | Cs3g21480 |
| CsTRXy1-2300-R | ACCATGGTGTCGACTCTAGATTGCTTGACGCTCAATGAATTCTC |  |  |
| CsTRXh1-qF | GCCGTTTCATTGCTCCTTTC | RT-qPCR | Cs1g24740 |
| CsTRXh1-qR | AGTCAGTGGCAACACTCTTC |  |  |
| CsTRXf1-qF | GATCACAGCCTCTGCTTTCT |  | Cs6g02830 |
| CsTRXf1-qR | TCGCCTTCTTCGCTACTAATG |  |  |
| CsTRXm1-qF | CCGGATGATCCACCCTATTATT |  | Cs1g07120 |
| CsTRXm1-qR | TGGGATGCTCCTAATTCCATATC |  |  |
| CsTRXo3-qF | CATGCAACCCTTGCTGAAAC |  | Cs4g13930 |
| CsTRXo3-qR | GCTTGACTCAGGGCTTCTAAA |  |  |
| CsTRXx2-qF | CCAGGAATACGGAGACAGATTG |  | Cs7g13660 |
| CsTRXx2-qR | TCAAGGTCGGCAATCCATAAA |  |  |
| CsTRXy1-qF | CTTGTCAGTACATGGCTCCTATC |  | Cs3g21480 |
| CsTRXy1-qR | AGTTGGCAAGGCCTCTATTC |  |  |
| GAPDH-qF | CAGCACTCAAAGGCAAACTAAA |  | Reference gene |
| GAPDH-qR | GGACACCTGAACGACAAGAT |  |  |
