# Supplementary material for: Systematic analysis of the thioredoxin gene family in Citrus sinensis: identification, phylogenetic analysis, and gene expression patterns
Source: Plant Signal Behav. 2023 Dec 17;18(1):2294426. doi: 10.1080/15592324.2023.2294426 (PMC10730155; doi:10.1080/15592324.2023.2294426)
Supplement: Table S2.docx [file KPSB_A_2294426_SM1105.docx]

Table S2. Physical and chemical analysis of thioredoxin protein properties in *Citrus sinensis*.

| Gene name | Locus ID | CDS (bp) | Amino acids | MW (kDa) | PI | GRAVY | Subcellular location | Typical (Yes/No) |
| --- | --- | --- | --- | --- | --- | --- | --- | --- |
| CsTRXm1 | Cs1g07120 | 546 | 181 | 19.70 | 10.01 | -0.046 | Chloroplast | Yes |
| CsTRXx1 | Cs1g16190 | 549 | 182 | 20.79 | 4.84 | -0.247 | Chloroplast | Yes |
| CsTRXh1 | Cs1g24740 | 363 | 120 | 13.21 | 5.19 | 0.087 | Cytoplasm | Yes |
| CsTRXh2 | Cs2g07350 | 384 | 127 | 14.12 | 4.45 | 0.091 | Cytoplasm | No |
| CsTRXo1 | Cs2g11490 | 576 | 191 | 21.22 | 9.92 | -0.194 | Chloroplast / Nucleus | Yes |
| CsTRXo2 | Cs2g12960 | 393 | 130 | 14.95 | 6.29 | -0.642 | Chloroplast / Cytoplasm / Nucleus | No |
| CsTRXm2 | Cs3g20630 | 549 | 182 | 19.91 | 8.67 | -0.034 | Chloroplast | Yes |
| CsTRXy1 | Cs3g21480 | 510 | 169 | 18.80 | 10.29 | -0.249 | Chloroplast | Yes |
| CsTRXh3 | Cs3g23540 | 513 | 170 | 19.12 | 7.99 | -0.476 | Nucleus | Yes |
| CsTRXh4 | Cs3g25420 | 372 | 123 | 13.48 | 4.96 | 0.067 | Cytoplasm | No |
| CsTRXh5 | Cs3g26690 | 1047 | 348 | 39.02 | 8.57 | -0.192 | Chloroplast | Yes |
| CsTRXh6 | Cs4g12290 | 387 | 128 | 14.31 | 8.76 | 0.053 | Cytoplasm | No |
| CsTRXo3 | Cs4g13930 | 696 | 231 | 25.49 | 8.75 | -0.122 | Chloroplast | No |
| CsTRXf1 | Cs6g02830 | 558 | 185 | 19.90 | 10.01 | -0.076 | Chloroplast | Yes |
| CsTRXh7 | Cs6g10260 | 420 | 139 | 15.45 | 4.65 | -0.224 | Cytoplasm | Yes |
| CsTRXh8 | Cs6g20580 | 432 | 143 | 16.00 | 6.1 | -0.327 | Cytoplasm | Yes |
| CsTRXh9 | Cs7g04520 | 390 | 129 | 14.87 | 8.67 | -0.326 | Cytoplasm | Yes |
| CsTRXx2 | Cs7g13660 | 579 | 192 | 21.45 | 8.47 | -0.122 | Chloroplast | Yes |
| CsTRXh10 | Cs7g29430 | 426 | 141 | 15.84 | 7.27 | -0.147 | Cytoplasm | No |
| CsTRXm3 | Cs8g06060 | 519 | 172 | 19.07 | 8.73 | -0.025 | Chloroplast | Yes |
| CsTRXh11 | orange1.1t00126 | 987 | 328 | 36.85 | 5.82 | -0.551 | Cytoplasm / Nucleus | Yes |
| CsTRXo4 | orange1.1t02220 | 570 | 189 | 21.92 | 9.64 | -0.393 | Chloroplast | No |
